# Cardiac involvement in laminopathies

**DOI:** 10.1186/1750-1172-10-S2-O25

**Published:** 2015-11-11

**Authors:** Giuseppe Boriani, Elena Biagini, Karim Wahbi, Denis Duboc

**Affiliations:** 1 Institute of Cardiology, Department of Experimental, Diagnostic and Specialty Medicine, University of Bologna, S. Orsola-Malpighi University Hospital, Bologna, Italy; 2 Service de cardiologie, Hopital Cochin, Paris, and Sorbonne Universités, UPMC Univ Paris 06, INSERM UMRS974, CNRS FRE3617, Center for Research in Myology, Paris, France

## 

Lamin A/C gene mutations can be associated with myocardial diseases, usually characterized by dilated cardiomyopathy and/or arrhythmic disorders. Phenotypic penetrance is age-related but expression is extremely heterogeneous, so that muscular and arrhythmic disease can be present in combination in the same patient, or one phenotypic manifestation can appear earlier than the other or even not become overt for a long time period[1]. From a cardiological point of view, aetiological diagnosis in dilated cardiomyopathy, and specifically the diagnosis of cardiolaminopathy, is relevant, since clinical and prognostic implications as well as specific management strategies can be different, particularly with regard to prevention of sudden cardiac death.

**Figure 1:**
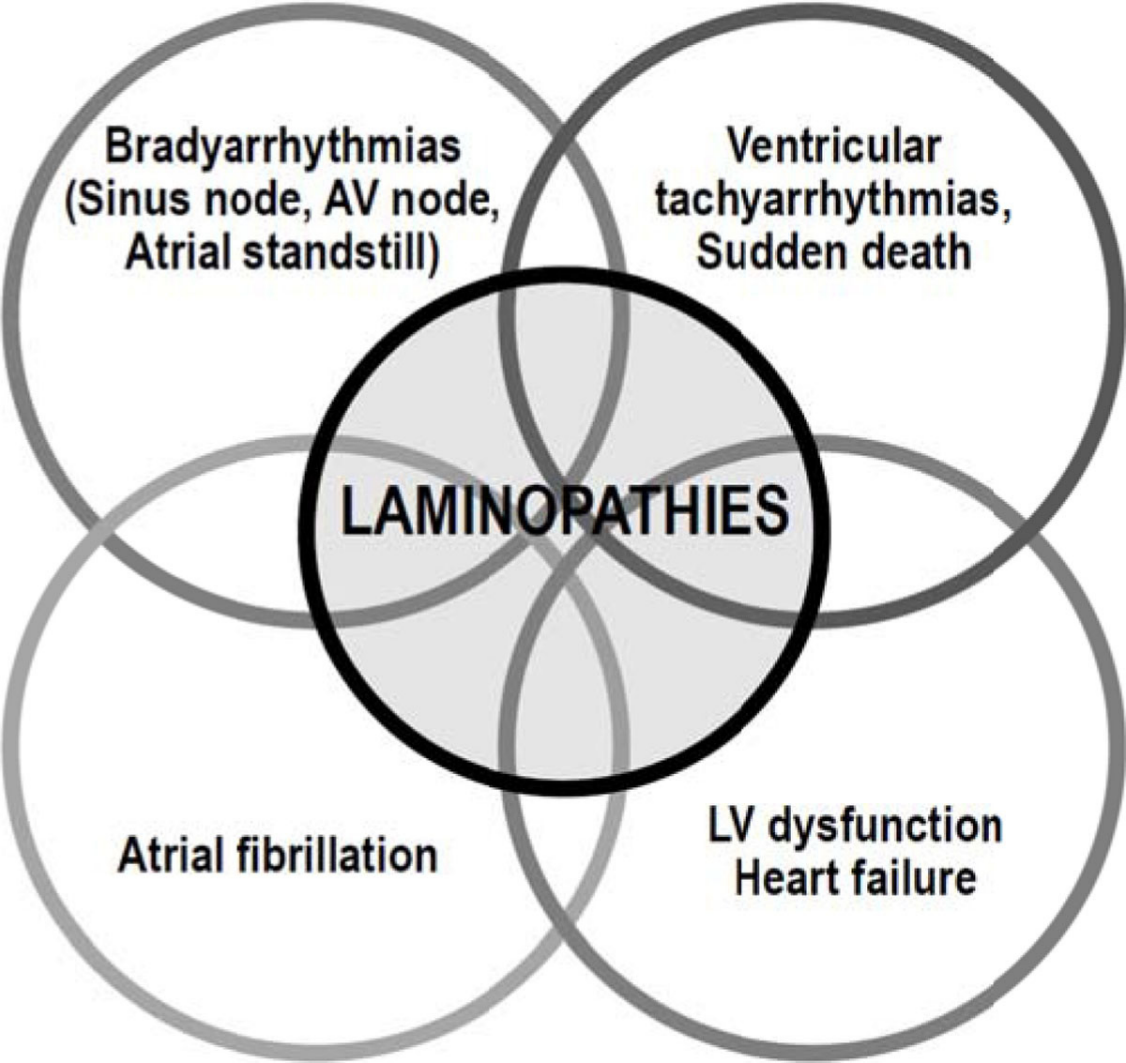
Spectrum of cardiac involvement in cardiolaminopathies, with regard to arrhythmic disturbances and heart failure. Legend: AV: atrio-ventricular, LV: left ventricular.

Patients can be diagnosed as being affected by a cardiolaminopathy as a result of a cardiological workup performed for symptoms of heart failure or for arrhythmic events or can be diagnosed incidentally or during family screening. Family history, physical examination, laboratory findings (specifically serum creatine kinase values) and ECG findings are important “red flags” to diagnose a cardiolaminopathy. Patients with cardiolaminopathies may present a wide range of arrhythmic disturbances, which include either bradyarrhythmias (conduction disturbances and ario-ventricular blocks, sinus node dysfunction, atrial standstill) or tachyarrhyhmias (atrial fibrillation, ventricular tachycardia and ventricular fibrillation), in variable combinations, and with frequent association with left ventricular dysfunction and heart failure (Figure 1). The presence and severity of arrhythmic disturbances is usually not related to the presence and degree of neuromuscular impairment[2-4]. The most common clinical manifestations are lightheadedness, syncope, palpitations, or even ischemic stroke due to cardioembolism (in case of atrial fibrillation or atrial standstill) or sudden death[2-5]. Implantation of a pacemaker protects form the consequences of bradyarrhythmias, while an implantable cardioverter defibrillator (ICD) is able to interrupt malignant ventricular tachyarrhythmias, thus preventing sudden cardiac death[6]. Biventricular pacing is a form of cardiac stimulation, referred as cardiac resynchronization therapy (CRT) that may improve cardiac function in case of heart failure, low ejection fraction and ventricular dyssynchrony[7]. Clinical decision making has to consider the risk and benefit of brady- and tachyarrhythmias, taking into account presence/absence of ventricular dysfunction, and the decision to implant a cardiac electrical device (pacemaker, ICD, with/without CRT) should consider potential risks and benefits (brignole EP). In a multicenter study a series of risk factors emerged as predictors of the occurrence of ventricular tachyarrhythmias (male gender, non-sustained ventricular tachycardia, left ventricular ejection fraction < 45% and non-missense mutation) and their presence or combination and should help for the decision to implant an ICD[8].
